# Combined Injection for Control of Iron-Deficiency Anemia and Coccidiosis in Piglets Decreases Stress at Management Time

**DOI:** 10.3390/ani14152241

**Published:** 2024-08-01

**Authors:** Daniel Sperling, María Rodríguez, Laura de Frutos, Joaquín Morales

**Affiliations:** 1 Ceva Santé Animale, 10 Avenue de la Ballastière, 33500 Libourne, France; 2 Animal Data Analytics, S.L., Dámaso Alonso 14, 40006 Segovia, Spain; maria.rodriguez@ada-animaldata.com (M.R.); laura.defrutos@ada-animaldata.com (L.d.F.); joaquin.morales@ada-animaldata.com (J.M.)

**Keywords:** piglets, welfare, iron-deficiency anemia, cystoisosporosis, parenteral

## Abstract

**Simple Summary:**

In this study, we sought to compare the effects of two treatment protocols for the control of iron-deficiency anemia and cystoisosporosis in piglets by studying the behavioral and physiological (stress) reactions in 3-day-old animals. In one protocol, intramuscular injection with a toltrazuril (TZL) and iron-based (gleptoferron) combination product was administered; in the other, the injection of iron was combined with an oral application of TZL. A total of 288 piglets were divided into three sex-balanced groups: 96 piglets in an oral + parenteral group (O + P); 96 piglets in a parenteral group, (P); and 96 untreated animals in a control group. The response of the piglets to manipulation was studied using previously described methodology. Using video recordings, an evaluator assigned scores to indicate flight reactions and both the intensity and frequency of vocalizations in the treated piglets. More intense responses (value 2) were recorded in piglets from the O + P group, indicated by increased reactions to manipulation and flight reactions (*p* < 0.05). In addition, piglets in the O + P group emitted more screams during administration than animals in the P group (*p* < 0.05). Differences in growth performance between the treatment and control animals were not observed (*p* > 0.05). In conclusion, the administration of a combination product reduced stress during administration, as indicated by reduced vocalizations and reactions to manipulation.

**Abstract:**

The aim of the present study was to assess the short-term behavioral and physiological responses of piglets to different treatment protocols for the control of iron-deficiency anemia (IDA) and cystoisosporosis. Piglets were treated with either (1) an injection of iron combined with an oral application of toltrazuril (TLZ) by drenching or (2) a combination injection of TZL + gleptoferron; the behavior of the piglets was then evaluated. For this study, 288 piglets were divided into three experimental groups: 96 piglets were kept untreated (control group); 96 piglets received an oral administration of a generic TZL-based anticoccidial agent (20 mg/kg BW) along with intramuscular administration of iron dextran (200 mg/mL; 1 mL/piglet) at the same handling (oral + parenteral group, O + P); and 96 piglets received an intramuscular application of the combination product (parenteral group, P). For each treated piglet, the total handling time, flight reaction, and the intensity and frequency of vocalizations were determined using the methodology described by Scollo et al. (2020). Piglets in the O + P group were found to emit more screams during treatment administration than animals in the P group (21.05% vs. 8.42% of animals; *p* < 0.05). Piglets in the O + P group reacted worse to manipulation and oral administration because a higher percentage of animals continued to fidget even after handling (32.63% vs. 12.63%; *p* < 0.05). Differences in growth performance between the groups were not observed in our study (*p* > 0.05). In conclusion, the administration of a combination product reduced stress during administration, as indicated by reduced vocalizations and reactions to manipulation.

## 1. Introduction

In recent years, an increasing amount of attention has been paid to the welfare of animals, including livestock species such as pigs, reflecting an increased awareness of ethical obligations to maintain humane environments for production animals. This trend is clearly reflected in the different regulations and standards now applied in EU countries, but it can also be observed in many other important swine-producing countries worldwide [1,2]. The importance of welfare in the swine industry is indicated by the number of recent studies that have specifically focused on the welfare of pigs, including the levels of stress experienced by animals [3]; indeed, along with dairy cows, pigs have been the most frequently investigated livestock animals in recent years (2015–2021) [3]. During this period, many European consumers have sought more animal-friendly products produced under higher welfare standards at the farm level, prompting the raising of concerns with respect to some pig production systems [4,5]. Researchers have found that many consumers are willing to pay more for products produced under higher animal-welfare standards [6].

Iron-deficiency anemia (IDA) and cystoisosporosis are the two main pathologies in pre-weaned piglets, and both occur worldwide [7,8]. In piglets, the coccidian parasite *Cystoisospora suis* is the causative agent of cystoisosporosis. This is the most frequent parasitic infection causing clinical diarrhea and production losses in piglets, with high prevalence rates reported in swine-producing countries. On pig farms where *Cystoisospora*-related diarrhea occurs, metaphylactic treatment with toltrazuril (TZL) is both recommended and frequently used to control infection not only in Europe but also in other important pig-producing countries [8,9]. In one recent study, it was reported that more than 60% of investigated farms used control programs based on the application of TZL for anticoccidial treatment during the first days of life, as part of commonly applied husbandry practices in farrowing houses [8]. In piglets, during the first 3 days of life (DOLs), single-dose intramuscular (IM) supplementation with 200 mg of a commercial formulation of iron (gleptoferron/dextran-based) is used for the prevention of IDA; indeed, all piglets produced under industrial farming conditions should be treated [7,10]. Until now, cystoisosporosis and IDA have been managed based on the oral application of TZL by drenching and intramuscular injections of iron complexes, administered separately. However, a new combination product containing TZL and iron (gleptoferron) for intramuscular application (Forceris^®^) has recently been registered for the metaphylaxis of both pathologies [11,12]. Both medical interventions rely upon early treatment during the first DOLs and both are considered to be part of standard veterinary management in farrowing houses.

These treatments are applied to piglets at a very early stage of life when numerous husbandry practices are also routinely applied; these include surgical castration, tail docking, and teeth clipping, as well as ear notching, tagging, or tattooing. In general, these are interventions that induce damage to sensitive tissues and may cause acute and/or chronic pain, affecting the welfare of the piglets. These issues might be addressed by a reduction in the number of interventions. Such a reduction might be of benefit not only for treated piglets but also the workers responsible for the management of animals in farrowing houses. It is well known that the amount of handling and restraint to which pigs are subjected in farrowing houses varies depending on the number and types of practices applied, and this variation is known to impact the behavior, physiology, and productivity of pigs [13].

In some published studies concerned with different vaccine-administration routes, the effects on animal welfare have been evaluated based on the impacts of procedures on behavioral parameters and on vocalization [1,14]. These investigations have revealed that piglets respond differently to different means of administration, with some vaccination techniques being more welfare friendly than others. To date, neither the per os application of pharmaceuticals nor the combination of such application with injectable forms of treatment have been widely discussed, and information is generally lacking in this area. However, one previous pilot study did reveal differences in the effects on piglet behavior resulting from to additional oral applications of TZL [15].

Different biomarkers can be used for the laboratory evaluation of stress in pigs. These biomarkers can be classified into different types and groups, and these may differ in elevation time according to the physiological system or axis evaluated: the sympathetic nervous system; the hypothalamic–pituitary–adrenal axis; the hypothalamic–pituitary–gonadal axis; or the immune system [16]. Acute-phase proteins (APPs) are blood proteins that characterize responses to inflammation and infection; they can also be used for the evaluation of physical or psychological stress [17]. Today, APPs serve as a tool for the diagnosis of complex diseases in pigs, but their role as stress biomarkers has also been studied in different animal species [18,19,20].

The aim of this study was to evaluate the effects of different treatment protocols used to control IDA and cystoisosporosis. To one group of piglets, a single injection with a combination product was administered; to another group, an injection of iron and an oral application of TZL by drenching was separately administered. Then, the effects on piglets in terms of pain response (vocalizations), behavior (flight reactions) and humoral stress response (levels of cortisol and major acute-phase protein in pigs (Pig-MAP)) was compared.

## 2. Materials and Methods

### 2.1. Animals and Facilities

The trial was carried out on a commercial farm (Centro de experimentación porcino) located in Aguilafuente (Segovia, Spain). A total of 288 piglets (Topigs TN70 sow × Pietrain boar) were used in the study. A total of 24 sows (Topigs TN70; third to sixth parturition) were selected, each with the same size of litter (12 piglets). The sows received a normal vaccination program, including vaccination against Porcine Parvovirus + Erysipelas (15 days after farrowing), Aujeszky’s disease (every 4 months), colibacillosis + *Clostridium perfringenst* type C (30 days before farrowing), and anthelmintics (applied in feed every 6 months). The sows and their offspring were all healthy at the start of the study, and no excessive manipulation, vaccinations, or treatments were applied to piglets during the study except for those set out in experimental protocols. Piglets were three days old at the start of the treatment application study (48–72 h after birth), and there were no differences between the initial body weights of the study groups (1.64 kg, *p* = 0.8253).

The lactation room had controlled environmental conditions (light/dark, 16L:8D; temperature, from 23 °C at farrowing to 20 °C at weaning; relative humidity, 60 to 75% by a forced ventilation system), and the nests of piglets were equipped with infrared light sources that allowed for a temperature between 32 °C and 28 °C to be maintained from farrowing to weaning.

All the experimental procedures were approved by the Animal Ethics Committee of the CEU Cardenal Herrera University (CEEA 22/018) and complied with the Spanish guidelines for the care and use of animals in research (BOE 2013), in agreement with European Union Regulation 2010/63/UE.

### 2.2. Experimental Design

#### 2.2.1. Animals and Treatments

Within the first 48 h after birth, the piglets were individually identified with ear tags and then weighed. The litters were equalized to 12 piglets/litter by cross-fostering between sows that farrowed on the same day. Piglets were kept in the same litters so that cross-fostering was avoided after day −1. The allocation of piglets to the treatment groups was carried out at random within each litter using a computer-generated random allocation procedure. The piglets were distributed into three experimental treatment groups with similar sex ratios. Hence, from each litter, 4 piglets were allocated to each of the treatment groups.

The experimental groups were established as follows: 96 piglets were left untreated, i.e., they received no treatment with iron/anticoccidial agents and no handling (control group, C); 96 piglets received an oral administration of generic anticoccidial agent by drenching (20 mg/kg BW; Baycox 5%, Elanco, Buenos Aires, Argentina) along with intramuscular administration of iron dextran (200 mg/mL; 1 mL/piglet; Calidex-G^®^; CALIER S.A., Barcelona, Spain) at the same handling (oral + parenteral group, O + P); finally, 96 piglets received an intramuscular application of the combination product (1.5 mL; Forceris^®^, CEVA Santé Animale, Libourne, France) (parenteral group, P).

The application procedures for each treatment (O + P or P) were performed by the same group of researchers, none of whom were involved in the analysis of the results.

#### 2.2.2. Behavioral Evaluations

To evaluate the behavior of the piglets, 2 days before the expected farrowing date, 24 double-infrared video cameras (Sricam^®^ SP017, Shenzhen Sricctv Technology Co., Ltd., Shenzhen, China) were installed in each farrowing box. Video information was captured and stored using a digital video recording system and an external memory drive. The cameras began recording 3 h before the start of the treatment session and the administration of the iron/anticoccidial supplementation. All video images were analyzed by two different observers using VLC software (version 3.0.12, VideoLAN Organization, Paris, France). The collected information from the video images was summarized in an Excel file by the two observers.. Vocalization was recorded at the time of treatment session by two different observers. When the collected data were analyzed and interobserver repeatability was confirmed, the observation was considered as valid.

During the application procedures, each piglet was gently held and restrained in the arms of an operator during treatment application. The total handling time was considered to be the exact period between the piglet being picked up, the treatment application, and its final release, and it was measured in seconds.

The response of the piglets to manipulation was then studied. For this purpose, the response of each animal at the moment when treatment was administered was evaluated using the methodology described by Scollo and colleagues [1]. Firstly, based on the video recording from the time of treatment application, an evaluator assigned a score to determine the flight reaction. A value of 0 was assigned when the piglet remained still and did not require efforts to restrain it. A value of 1 was assigned when the piglet moved at the time of vaccination/oral administration by drenching, but afterwards remained still. Finally, a value of 2 was assigned when the piglet continued to fidget even after handling had ceased. Each piglet was gently held and restrained in the arms of the operator during application procedures, and all treatments were administered by the same experienced individual.

The vocalizations of piglets were assessed by two different evaluators on the basis of direct observation, not video recordings. A value of 0 was assigned when there were no vocalizations or when only brief grunts were emitted. A value of 1 was assigned when there were repeated grunts but no more than one scream was emitted. Finally, a value of 2 was assigned when at least two screams were emitted.

#### 2.2.3. Biological Analysis

**Figure 1:**
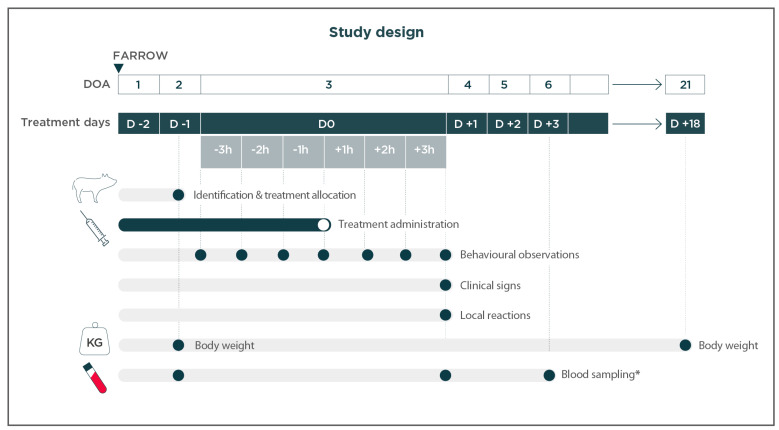
Study schedule outlining timeline and time points (ticked boxes) of study procedures, expressed as days of age (DOA) and days (D). (*): One blood sample of 5 mL (to obtain serum) in 2 animals per litter (O + P and P groups in each litter) were taken. The election of piglets was conducted at random. The same piglets were sampled. DOA—day of age; D—day; h—hour; Kg—kilogram.

Blood was sampled from 1 piglet/litter in each of the treatment groups O + P and P (2 piglets per litter, 24 piglets/treatment group: 48 piglets in total) at the following timepoints: on day 0; after the video recording (3 h after treatment administration); and on day 3. Piglets from the C group were not sampled. The baseline status was analyzed from samples collected from piglets the day before treatment (day −1). Cortisol was analyzed on day 0 to detect the acute stress response in piglets. PigMAP is a nonspecific biomarker used to quantify inflammation (potential injury response) and/or stress. PigMAP levels are known to remain elevated even after stressful activities cease [19]; hence, this was analyzed on day 3 Figure 1.

The concentration of cortisol in plasma samples was analyzed using the competitive-binding ELISA method with a commercial ELISA kit (DRG^®^ Cortisol Enzyme Immunoassay kit, DRG^®^ Instruments, Marburg, Germany). The assay ranged from 0 to 800 ng/mL, the sensitivity was determined to be 2.5 ng/mL, and the inter-assay and intra-assay variation coefficients were 8.1% and 6.6%, respectively. The concentration of PigMAP in plasma samples was analyzed using the immunoturbidimetric method (Turvovet PigMAP kit, Acuvet Biotech, Zaragoza, Spain). The assay ranged from 0 to 5 mg/mL, the limit of detection was 0.0048 mg/mL, and the inter-assay and intra-assay variation coefficients were 3.5% and 7.3%, respectively.

#### 2.2.4. Performance Evaluation

Piglets were weighed individually the day before treatment (day −1) and on day 18 of study (day 21 of lactation). The average daily gain (ADG) of the piglets was calculated for this period (from day −1 to day 18).

### 2.3. Statistical Analysis

Statistical analysis was performed using R software (version 4.1.2). The percentages of different scores (values of 0, 1, and 2) for both flight reaction and vocalization were analyzed using a proportion test. After an initial normality and homoscedasticity test, the length of handling time was analyzed using a sign test, the concentrations of cortisol and PigMAP were analyzed using ANOVA, and performance parameters were analyzed using a Kruskal–Wallis test. Least squares means (LS means) were computed for each. Finally, a significance level of *p* < 0.05 was applied.

## 3. Results

As expected, the use of different procedures of application of iron/anticoccidial supplementation was found to affect the handling times (mean ± SEM), with these being longer in the O + P (10.54 ± 0.21 s/piglet) group than in the P group (4.36 s ± 0.14 s/piglet) (*p* < 0.05). As piglets from the C group (control) were not handled, no handling times were recorded for animals in this group.

In terms of management during treatment administration, no piglet was assigned a 0 value for response to manipulation, neither for flight reaction nor vocalization. None of the treated piglets remained still, and all of them produced noise to some extent.

An evaluation of the piglets that reacted to manipulation and to the treatment procedure in terms of behavioral observations and vocalization is shown in Table 1. In relation to the flight reaction, there were no significant differences between treatments for the piglets that moved at the time of treatment (value 1 on the scale) (*p* > 0.05), neither were there any significant differences between treatments when considering piglets assigned a value of 1 on the scale of vocalization, i.e., animals that repeatedly grunted and/or emitted a maximum of one scream (*p* > 0.05).

**Figure 2:**
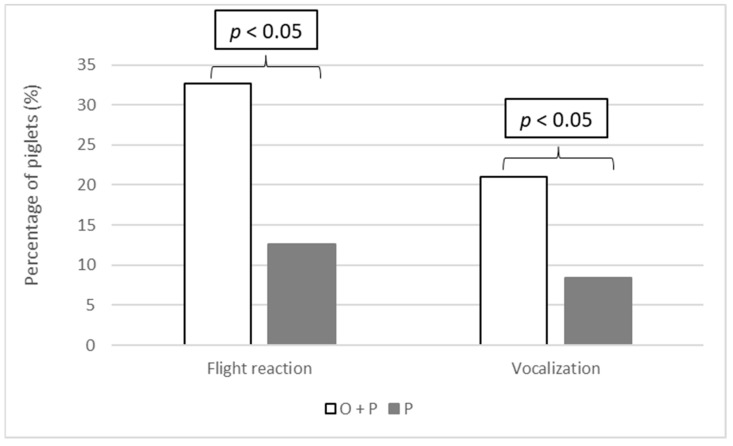
Percentages of piglets assigned a value of 2 on the scale of evaluation of manipulation during the application of treatment, considering both flight reactions (piglet continued to fidget even after handling had ceased) and vocalizations (at least two screams were emitted) in oral + parenteral (O + P) and parenteral (P) groups.

Upon evaluating piglets with values of 2 on the scale response to manipulation, in terms of both flight reaction and vocalization, a significant difference was observed between groups. Piglets in the O + P group reacted more intensely to manipulation and exhibited longer and more intense flight reactions because a higher percentage of animals continued to fidget compared with group P (*p* < 0.05) Moreover, piglets from the O + P group emitted more screams during administration than those in the P group (*p* < 0.05) (Figure 2 and Table S1).

Turning to blood analysis, although there were no statistical differences in terms of the cortisol concentration (*p* = 0.5133) and increase from baseline (*p* = 1.0000), the PigMAP concentration tended to be lower in the P group than in the O + P group (0.98 ± 0.36 vs. 1.31 ± 0.99 mg/mL, respectively; *p* = 0.1016), with a highly significant increase on day 3 taking into consideration the baseline status (*p* = 0.0004) (Table 2).

Treatment administration did not affect (*p* > 0.05) growth performance during lactation; consequently, the body weight on day 18 was similar among experimental groups (Table 3).

## 4. Discussion

The metaphylactic treatment of cystoisosporosis and the application of iron for the prevention of IDA are both part of the standard management of piglets during the first days of life. A recently introduced treatment option that combines both interventions (a combination injection) may reduce the time required for the procedure, avoid the need for deep oral drenching, and consequently reduce stress- and pain-related behavior in piglets.

In the present study, the behavioral response, as measured by flight reaction and its intensity, was evaluated. Our results suggest that a treatment protocol involving an injection of iron and an additional application of TZL administered orally by drenching resulted in higher levels of fear-related activity. The main difference observed was an increased frequency of flight reactions, with more piglets continuing to fidget after the procedure. Domestic pigs are typically fearful of humans to some degree, and they often exhibit avoidance behaviors that are stressful, similarly to wild pigs [13,21]. The intensity of such stress may be affected by the levels of pain associated with treatment procedures and the lengths of time for which piglets are required to be restrained. Previously, researchers have evaluated the responses of piglets to different vaccination techniques through the assessment of flight reactions and found that variations in the levels of discomfort experienced by animals may result from differences in the restraint times and in the types of inoculation used (i.m. application vs. intradermal); both of these have been shown to affect the levels of tension [1]. The use of a needle and intramuscular application has been shown to induce different levels of response in terms of flight reactions and the emission of screams not only in most piglets but also in other categories of pigs (sows). When intramuscular application is combined with other procedures, such as oral application of medication, the level of stress response in piglets is increased, as indicated by the results of the present study.

Differences in the responses among piglets may also be attributed to the time required for the handling of animals, which is twice as long when piglets are injected and orally drenched (*p* < 0.05). Typically, pigs experience handling and restraint only a few times in the course of their life, and such experiences are usually associated with low-grade pain because of invasive treatment procedures. In fact, it has long been recognized that handling and manipulation impact the behavior, physiology, and productivity of pigs [22,23]. Consequently, any combined procedure that limits the time needed for manipulation may be considered useful. Previous studies of piglets immediately after weaning have found that different types of exposure (rough, gentle, or minimal handling) impact the emotional state of the animals and influence their cognitive abilities [22]. The intensity of the response to other pain-associated procedures is probably related not only to the type of procedure employed but also to the length of time required for the procedure to be carried out, as has been demonstrated for short-duration procedures such as ear tagging when compared with other techniques [22].

The number of vocalizations and their intensity have frequently been used as an identification method in studies on the welfare of pigs, sometimes in combination with other behavioral and physiological responses, mainly due to potential differences in the sensitivity of vocalization to different techniques [24]. In the present study, the differences observed between the treatment groups supported the results of the flight test, in that a greater intensity of response was recorded in the O + P group of piglets. One limitation of the present study was that the exact frequency of vocalization was not evaluated; despite this, a significantly more intense response was recorded by two different evaluators, suggesting a possible effect of additional treatment on piglets. A limitation of such approach is fact, in that the verification of the data collected for accuracy is not possible when treatment session is finished. With regard to vocalization frequency, some controversial results have previously been reported [1,25]. However, a similar approach involving a comparison of grunts or squeals has been successfully used previously, supporting the idea that piglet screams seem to have a particular position in the call repertoire, expressing stress or pain [25].

The results of the present study suggest that a single combination injection is more welfare friendly to piglets. In terms of both the behavioral parameters considered (escape attempts and vocalization), negative expression was significantly more prevalent in the O + P group of piglets.

Oral drenching may be considered an especially stressful means of administering treatment to piglets. The administration of oral drugs can be challenging, as it requires a practitioner to place the medication at the base, or far back, of the animal’s tongue and then close its mouth to ensure that the medication is not lost. In general, dosing compliance with respect to oral veterinary formulations is also considered an important issue in other species [26]. The palatability of a medication can significantly impact the dosing compliance, with “palatable” typically defined as a product that is pleasant or acceptable to taste and, hence, fit to eat or drink. However, although this definition stresses the role of taste, it ignores the role of post-ingestive feedback in determining how an animal evaluates the hedonic value of a particular taste [27].

In the field of swine medicine, information on this subject is generally limited. Further studies on the oral supplementation of pharmaceuticals should focus on the palatability of medication, because variations in taste can impact stress responses in piglets. In a previous study of piglets with light body weights, sham drenching was found to have no effect on production parameters such as live body weight or mortality, either during the drenching period, the suckling period, or after weaning; however, this study did not focus on specific welfare impacts [28]. Using drenching in such situations, like in the supplementation of colostrum to light piglets to improve their survivability or in the case of vaccination with live vaccines when no alternative route of administration is available, fully justifies any possible stress arising from catching and restraining animals.

In the present study, two markers were selected for the humoral assessment of stress response: (1) pig major acute-phase protein (Pig-MAP), as a representative of a group of acute-phase proteins that are specific to pigs, and (2) cortisol, which is widely used as a stress marker in animals. Before the start of the study (D−1), the concentrations of the two markers did not differ in the piglets, suggesting a similar status (*p* = 0.40 and *p* = 0.23, respectively). Indeed, the concentration of plasma cortisol did not differ between the groups. Previous results obtained by the authors only indicated a trend for a higher level of cortisol in the O + P group in a pilot trial with a limited number of animals [15]. Similarly, and in line with the present study, the authors of [14] found no significant effect in the assessment of different administration techniques of vaccination in piglets at weaning based on basal cortisol levels in saliva samples (*p* > 0.05) [14]. One possible explanation is that this specific marker is not sensitive enough for the differentiation of low-pain interventions. Other markers of humoral response might be more suitable for this purpose [1,29]. Another possible influence is the timing of sample collection previously reported.

Pig-MAP has previously been described as a plasma protein that is the pig counterpart of human serum protein PK-120 substrate for plasma kallikrein (PK); it has shown promise as a good marker of pathologies, health, and welfare in pigs [30]. Recent studies have supported the idea that analyzing Pig-MAP in pigs might be a useful tool in routine health and welfare monitoring, in part due to its suitable pharmacokinetics profile (pK) [20,31].

In the present study, a trend was observed in the PigMAP concentration, with a significant increase, taking into consideration the baseline status, in the O + P group. The concentration of PigMAP marker on the Day−1 was only numerically higher in the P group, comparable with the other group. A previous analysis of this marker in physiological situations considered as stressful (sows at parturition and weaned piglets) showed elevated levels, similar to those obtained in piglets injected and orally drenched by TZL at the same time. Compared to proposed interpretive criteria for healthy pigs, 0.5 mg/mL (blood), both obtained average values, suggesting a certain level of stress associated with manipulation and the administration of products, but with significantly higher trend observed in O + P group.

The potential use of Pig-MAP protein as a stress marker and welfare indicator in piglets is not yet fully understood, and further exploration is needed in this area. However, the results of this research suggest that it might be considered a stress marker in addition to those previously described. One possible benefit of the Pig-MAP biomarker compared with the majority of stress markers may result from its apparent stability during the daytime (as recorded in saliva samples), when significant variability for other such markers in pigs has been reported, as well as the lack of any influence of sex upon its level [31].

Differences in zootechnical performances between the groups were not observed in our study. The early processing of piglets, including potentially painful and stressful procedures, may affect the piglets a few days after the procedure [32]. Its impact on the weaning weight is less obvious, and results, however, are not always consistent in the literature [32]. For that reason, a period of 18 days is probably too long to observe potential effects on the growth of piglets, and shorter, more frequent intervals may be applied in future studies.

## 5. Conclusions

In conclusion, in the present study, we found that administering a combination-product injection significantly decreased the time needed for the restraint and handling of animals compared with separate treatments of oral drenching and intramuscular injection. Based on an evaluation of the behavioral indicators, we found that a single injection for the prevention of IDA and cystoisosporosis decreased the stress levels during administration, as indicated by reduced vocalizations and less intense reactions to manipulation (flight reactions) in the post-treatment period. Finally, the lower PigMAP concentrations in animals injected with the combination product suggest that this marker might be considered for use along with other previously described stress markers.

## Figures and Tables

**Table 1:** Percentages of piglets with different responses (different values on the scale) to manipulation, considering flight reaction and vocalization observed in oral + parenteral (O + P) and parenteral (P) groups.

	Flight Reaction ^1^		Vocalization ^2^	
Treatment	% Value 1	% Value 2	% Value 1	% Value 2
O + P	48.42	32.63	67.37	21.05
P	55.79	12.63	70.53	8.42
*p*-value	0.3094	0.0010	0.6381	0.0141

^1^ Flight reaction: value 1—piglet moved only at the time of vaccination; value 2—piglet continued to fidget even after handling. ^2^ Vocalization: value 1—repeated grunts with a maximum of one scream emitted; value 2—at least two screams emitted.

**Table 2:** Concentrations of cortisol and PigMAP in plasma samples before the manipulation (day −1) and after (day 0 for Cortisol and day 3 for PigMAP), with variations from baseline determined in oral + parenteral (O + P) and parenteral (P) groups.

		Cortisol (ng/mL)			PigMAP (mg/mL)	
	Day −1	Day 0 *	Variation −1 and 0	Day −1	Day 3 *	Variation −1 and 3
O + P	80.46	66.50	−17.91%	0.803	1.314	63.64%
P	69.01	59.20	−14.22%	0.929	0.984	5.92%
*SEM*	6.68	5.84		0.054	0.098	
*p*-value	0.4046	0.5133	1.0000	0.2320	0.1016	0.0004

SEM: standard error of the mean. * Concentration on day −1 was included as covariate in the statistical analysis.

**Table 3:** Body weight (kg) and average daily gain (kg/day) of piglets before the manipulation (day −1) and after (day 18) in control (C), oral + parenteral (O + P), and parenteral (P) groups.

Treatment	Body Weight Day −1 (kg)	Body Weight Day 18 (kg)	Average Daily Gain (−1 to 18) (kg/day)
C	1.63	6.89	0.276
O + P	1.64	6.67	0.264
P	1.64	6.71	0.268
*SEM*	0.032	0.124	0.005
*p*-value	0.8253	0.6678	0.5471

SEM: standard error of the mean.

## Data Availability

Data are available upon reasonable request.

## Supplementary Materials

The following supporting information can be downloaded at: https://www.mdpi.com/article/10.3390/ani14152241/s1, Table S1: Response to manipulation and vocalization evaluation.
